# Single hydrogen atom manipulation for reversible deprotonation of water on a rutile TiO_2_ (110) surface

**DOI:** 10.1038/s42004-020-00444-4

**Published:** 2021-01-19

**Authors:** Yuuki Adachi, Hongqian Sang, Yasuhiro Sugawara, Yan Jun Li

**Affiliations:** 1 Department of Applied Physics, Osaka University, 2-1 Yamadaoka, Suita, Osaka, 565-0871 Japan; 2 Institute for Interdisciplinary Research, Jianghan University, 430056 Wuhan, China

**Keywords:** Structural properties, Mechanical properties, Scanning probe microscopy, Hydrogen fuel, Scanning probe microscopy

## Abstract

The discovery of hydrogen atoms on the TiO_2_ surface is crucial for many practical applications, including photocatalytic water splitting. Electronically activating interfacial hydrogen atoms on the TiO_2_ surface is a common way to control their reactivity. Modulating the potential landscape is another way, but dedicated studies for such an activation are limited. Here we show the single hydrogen atom manipulation, and on-surface facilitated water deprotonation process on a rutile TiO_2_ (110) surface using low temperature atomic force microscopy and Kelvin probe force spectroscopy. The configuration of the hydrogen atom is manipulated on this surface step by step using the local field. Furthermore, we quantify the force needed to relocate the hydrogen atom on this surface using force spectroscopy and density functional theory. Reliable control of hydrogen atoms provides a new mechanistic insight of the water molecules on a metal oxide surface.

## Abstract (web summary)

Rutile TiO_2_ is a prominent photocatalyst for overall water splitting, but the on-surface activation of hydrogen atoms is still not fully understood. Here, the authors use atomic force and kelvin probe force microscopy to study the lateral manipulation of hydrogen on a rutile (110) surface.

## Introduction

The hydrogen atom detection on a rutile TiO_2_ surface is an important topic owing to its intriguing chemical and physical properties related to atomistic water splitting and hydroxyl production on this surface^1–8^. Detail understanding of this adsorbate and its control at atomistic level is essential for fully elucidating the nature of a deprotonated water reaction on a TiO_2_ surface^1–23^. Moreover, oxidation of hydrogen atoms by oxygen molecule on the rutile TiO_2_ surface results in the reactive oxygen species (ROS), such as reaction intermediates of water species H_2_O, HO_2_, H_2_O_2_, and H_3_O_2_^24,25^. Exploring these species requires the fundamental understanding and detailed description of the interaction between oxygen and hydrogen atoms. Ideally, attacking these problems require to access the atomic scale study of a hydrogen atom on this surface, which remains a great challenge owing to the light mass and small size of the hydrogen atom. In particular, hydrogen species on a rutile TiO_2_ surface were investigated using various experimental techniques, including scanning tunneling microscopy (STM)^1–6,10–22,25,26^. STM provides a unique opportunity for electronically inducing the reactions of a hydrogen atom on this surfaces^3,10,18,21^. However, STM easily induces the stochastic behavior of molecules owing to its flowing current; therefore, it might be difficult to precisely control in the desired configuration at atomic level^3,18^. Moreover, the contrast mechanism of STM is related to the density of electronic states, which is still obscure to investigate the real-space of the atomic configuration^1,12,26^.

Atomic force microscopy (AFM), as a viable alternative, has been used to provide a precise measurement of the surface configuration, also manipulating atoms and molecules based on its force modulation mechanism^27–44^. Another fascinating capability of AFM is the possibility to measure directly the interaction forces that induce the adsorbate’s motion and thus, to provide a detailed insight into the interaction force for the target species^35–43^. Kelvin probe force spectroscopy (KPFS), owing to its force modulation mechanism, allows us to precisely control the different states of on-surface species signaled by the appearance of a jump in the frequency shift (Δ*f*) vs. bias voltage (*V*_bias_) parabola^9,27,28,31–33^. The vertical shift between the two parabolas is strongly influenced by the different local electric fields automatically formed between the tunneling junction^9,27,28,31–33^. Especially on the metallic surface, controllable on-surface lateral manipulation of the single molecule^45–47^ has been well established and the adsorbate can also be activated^48^ by means of electric field^49,50^. Moreover, on the rutile TiO_2_ surface, the vertical desorption of hydrogen atom^9,10,16,18,21^, reversible migration of hydrogen atom^23^, the stochastic motion of hydrogen atom induced by inelastic tunneling electrons^3^, manipulation of oxygen adatom^27^ and lateral tip induced excitation of the single molecule^35^ have been clarified. However, the lateral manipulation of the single hydrogen atom to the desired position on rutile TiO_2_ surface has been poorly reported experimentally up to now^10^, although it could provide an efficient means of the arrangement of the on-surface water splitting reactions, dissociation dynamics, which critically control the efficiency of heterogeneous catalytic reactions on this surface^1–23^. Especially, elucidating the interaction force between tip and water configuration on the TiO_2_ surface is important for answering the current controversial question of whether a proton atom should remain as one water molecule or two distinct hydroxyls from the mechanical point of view^1–8^. For local chemistry, the mechanical sensitivity and stability of hydrogen bond^40–43^ are also crucial properties, but studies of these properties remain elusive on this surface.

Here, we use AFM and KPFS to present the manipulation of hydrogen atom and reversible water reaction on a rutile TiO_2_(110)−(1 × 1) surface, and investigate its mechanical properties by the force field mapping. We analysis the manipulation outcome with the local electric field and the density functional theory (DFT). Our results demonstrate that the hydrogen atom can be manipulated along the oxygen row. The force field on the top of these configurations quantifies the possible tilting of the hydrogen atom on these configurations.

## Results and discussion

### Control of reversible deprotonation of water on a rutile TiO_2_ (110) surface

**Fig. 1:**
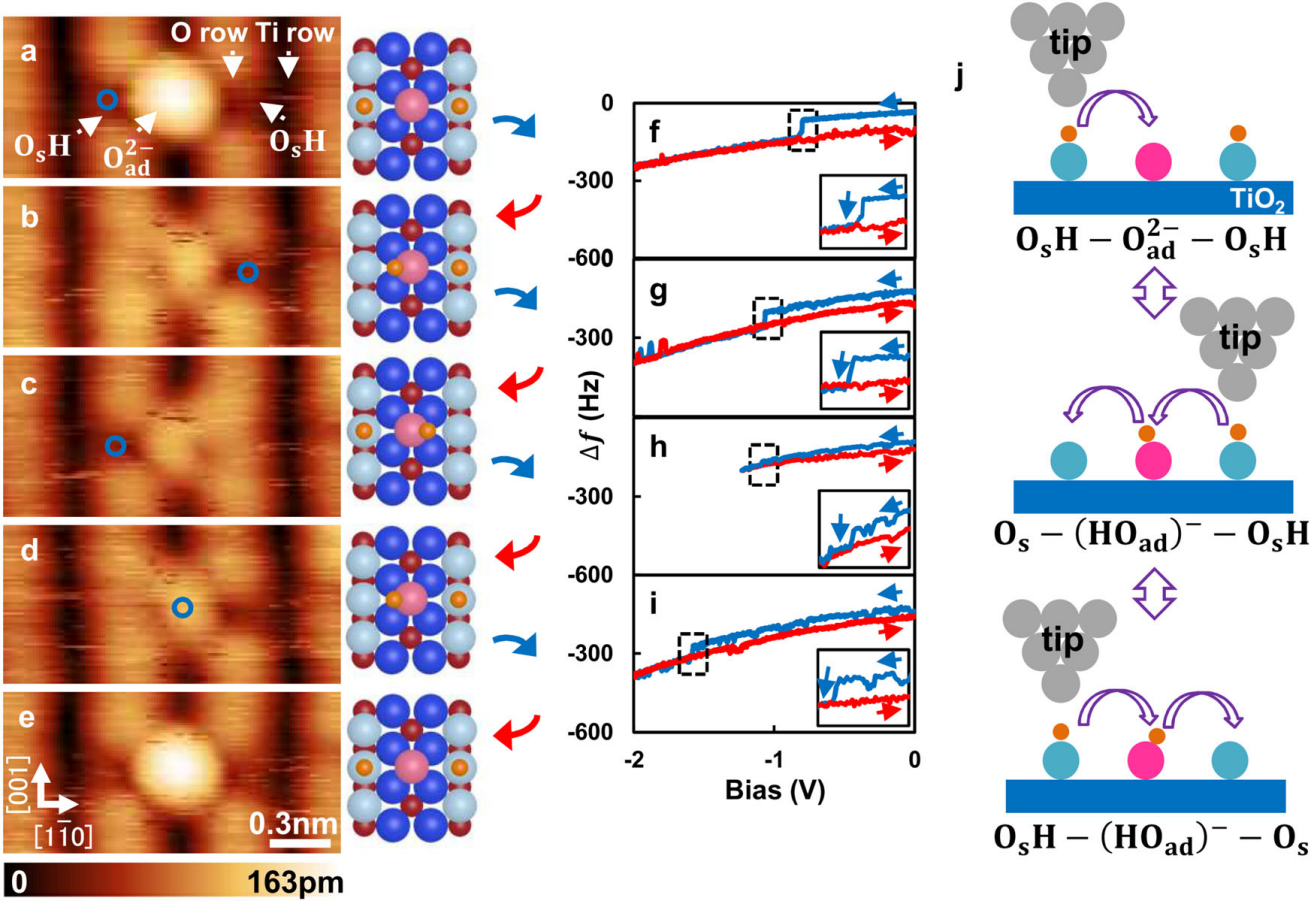
Control of O_s_H−O_ad_^2^−O_s_H reaction by KPFS manipulation. **a** AFM image of O_s_H−O_ad_^2−^−O_s_H on rutile TiO_2_ surface. **b**–**e** Consecutive AFM images of three adsorption species in the reversible manipulation sequence. Imaging parameters for **a**–**e**: constant Δ*f* mode, *V*_bias_ = 0 V and 1.0 × 2.0 nm^2^. The schematic of the figure, O_s_ (white balls): rows of twofold-coordinated bridge oxygen atoms; (blue balls): in plane threefold coordinated oxygen atoms; Ti (red balls at the middle of the oxygen row): rows of fivefold-coordinated Ti atoms; H (orange) hydrogen and O_ad_ (pink) adsorbed single-oxygen adatom. **f**–**i** Results of KPFS applied to images between **a**–**b**, **b**–**c**, **c**–**d**, and **d**–**e**. The feedback loop was switched off during the measurements, and the sample bias was ramped from zero to a certain negative voltage and then ramped back to zero. The blue and red lines show the spectra KPFS obtained by forward and backward directions, respectively. The tip position is indicated by a blue circle in the images of **a**–**d**. The insets in **f**–**i** show enlarged KPFS curves within dashed boxes placed around the regions of frequency jumps: between −1.0 V and −0.8 V (**f**), −1.1 V and −1.0 V (**g**), −1.2 V and −0.9 V (**h**) and −1.6 V and −1.5 V (**i**). **j** Schematic of O_s_H−O_ad_^2−^−O_s_H water reaction controlled by KPFS manipulation, including O_s_H−O_ad_^2−^−O_s_H, O_s_−(O_ad_H)^−^−O_s_H and O_s_H−(O_ad_H)^−^−O_s_.

First, we show an experiment for the reversible water reaction on the TiO_2_ surface. By using the KPFS technique, we experimentally demonstrated the water reaction as shown in Fig. 1(a–i). A Rutile TiO_2_ (110)−(1 × 1) surface contains twofold-coordinated surface bridging oxygen (O_s_) atoms and fivefold-coordinated titanium (Ti) atoms that are alternatively aligned^1^. Moreover, the practical sample preparation in ultrahigh vacuum (UHV) will induce point defects, such as oxygen vacancies (O_V_) and hydroxyl defects (O_s_H)^1^. When the TiO_2_ surface is exposed to oxygen at room temperature, oxygen will dissociate on this surface and will be adsorbed as an adatom (O_ad_) on Ti row^28,29^. We previously discovered that the O_ad_ has two stable charge states, namely, singly charged (O_ad_^−^) and doubly charged (O_ad_^2−^). Moreover, we previously found that O_ad_^2−^ is the most stable on the rutile TiO_2_ surface^28,29^. Figure 1(a) shows an atomically resolved AFM image of O_s_H−O_ad_^2−^−O_s_H species initially formed on top of the rutile TiO_2_ (110) surface obtained by the tip in hole mode (see also Supplementary Fig. S1). The two hydrogen atoms form a water configuration including O_ad_^2−^ and the adjacent O_s_ atoms. The characteristics of a net positively charged hydrogen atom were previously well accepted by various experimental techniques^1–23,26,30,34,44^. Two black spots that correspond to bistable O_s_H defects can be observed in Fig. 1(a)^9,30,34,44^. Notably, the oxygen adatom is spontaneously charged to O_ad_^2−^ and adsorbed between two O_s_H defects^28,29^. After AFM imaging (Fig. 1(a)), the tip was moved on top of the O_s_H defect and the bias was ramped from zero to a certain negative voltage and then back to zero (Fig. 1(f)). Figure 1(b) shows the AFM image of the same scan area at 0 V obtained immediately after the bias voltage back to zero, showing that the black spot corresponding to the O_s_H defect disappeared and O_ad_^2−^ became less bright. The characterization of this species is based on the movement of the target hydrogen atom toward O_ad_^2−^, inducing the formation of (O_ad_H)^−^, while the symmetric configuration is initiated by positioning the tip on top of the hydrogen atom and ramping the bias from zero to a certain negative voltage and then back to zero (Fig. 1(b) and (g)). Notably, this manipulation cycle is based on the double hopping of the hydrogen atom along the $[1\bar 10]$ direction as shown in Fig. 1(j). Hence, the manipulation cycle can be performed repeatedly as shown in the order of Fig. 1(b) → 1(g) → 1(c) → 1(h) → 1(d). Comparing with previous STM results^1–6,10–22,25^ and photoemission experiment^7,8^, one would expect to observe the O_s_−(H_2_O_ad_)−O_s_ configuration during this manipulation cycle. However, even though we bring back the bias voltage to zero immediately after the Δ*f* jump, O_s_H−(O_ad_H)^−^−O_s_ is generally transformed to O_s_−(O_ad_H)^−^−O_s_H (Fig. 1(c) → 1(h) → 1(d)). In reverse, this experimental result indicates that the O_s_−(H_2_O_ad_)−O_s_ configuration may be stochastically very rare compared with the O_s_−(O_ad_H)^−^−O_s_H configuration during this manipulation, possibly because of the low temperature of 78 K^5–8^ and the energy barrier for the recombination of two hydroxyls to water is relatively high while the barrier to move them is low owing to the DFT calculations in Du et al. study^5^. Additionally, considering the charged nature of the protons, it is not surprising that forming a water molecule is much less likely than moving both hydroxyls in concert. Hence, the transition of O_s_−(O_ad_H)^−^−O_s_H → O_s_H−(O_ad_H)^−^−O_s_ may generally occur immediately after the Δ*f* jump. Here, we note that even though we always placed the tip same height with symmetrically on the O_s_H−(O_ad_H)^−^−O_s_ configuration, we found that the bias voltage of Δ*f* jump slightly fluctuates within a bias range of ±0.15 V as shown in Fig. 1(f–h). We assigned this observation to different mesoscopic properties of tip-sample junction such as the very small tip-sample distance difference^27,28,32^, existence of intrinsic subsurface defect^44^ or polaron underneath the surface^51–53^. Notably, we do not observe the apparent telegraph noise between O_s_−(O_ad_H)^−^−O_s_H and O_s_H−(O_ad_H)^−^−O_s_ in KPFS measurement, thereby we safely exclude the possibility of inelastic tunneling electrons mechanism, which reported by Tan et al.^3^. The initial state shown in Figure 1(a) can be recovered by positioning the tip on top of (O_ad_H)^−^ and ramped the bias from zero to a certain negative voltage and then back to zero (Fig. 1(d) → 1(i) → 1(e)). Hence, a complete hydrogen manipulation cycle was successfully achieved: O_s_H−O_ad_^2−^−O_s_H ↔ O_s_−(O_ad_H)^−^−O_s_H ↔ O_s_H−(O_ad_H)^−^−O_s_.

**Fig. 2:**
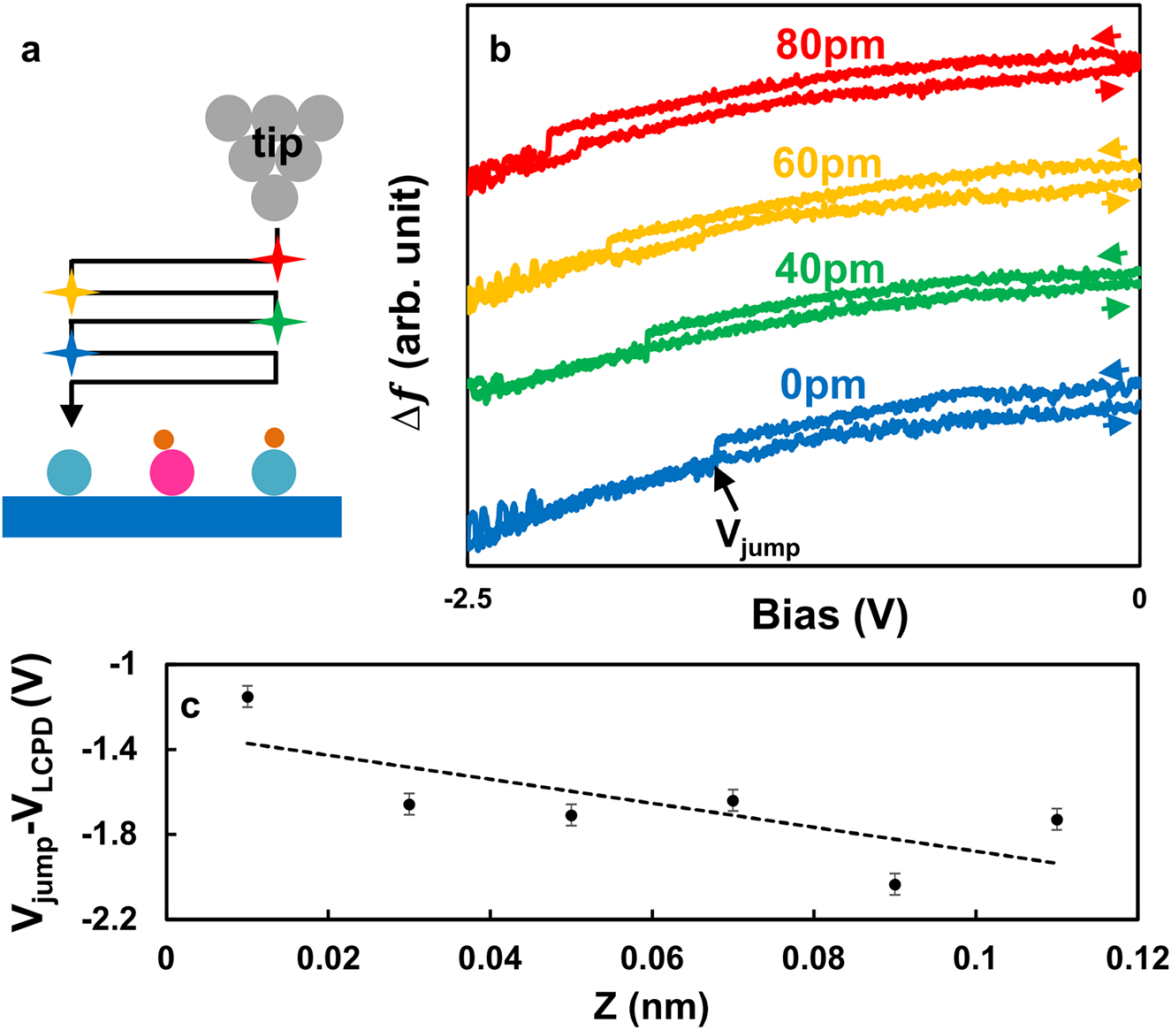
Distance dependence of KPFS measured on top of O_s_−(O_ad_H)^−^−O_s_H. **a** Schematic experimental procedure of distance dependence of KPFS. The black arrow indicates the tip path and the star mark shows the position where KPFS was performed. **b** KPFS taken at different tip heights, shown relative to the spectrum in blue, that corresponds to the closest tip heights considered. **c** The vertical axis is the subtraction of bias voltage where the frequency shift jump occurred in KPFS (*V*_jump_) from the local contact potential difference (*V*_LCPD_) obtained from the upper parabola in KPFS. The lateral axis is the relative distance from the sample. The black dotted line is a linear fitting to the data. The slope is −5.6 V/nm. The error bar indicates the deviation of the bias voltage in *V*_jump_.

To find out more physical mechanism behind the reversible manipulation between O_s_H−(O_ad_H)^−^−O_s_ and O_s_−(O_ad_H)^−^−O_s_H, we further perform KPFS measurement as a function of tip-sample distance, ensuring that no tip changes or deformation of the target O_s_−(O_ad_H)^−^−O_s_H could occur^9,27,28,31–33^. Fig. 2(a) shows the schematic of the experiment. The experiment was performed as follows. First, O_s_−(O_ad_H)^−^−O_s_H was prepared by the same procedure as shown in Fig. 1(a) → 1(f) → 1(b). Next, the initial position of the O_s_H in O_s_−(O_ad_H)^−^−O_s_H was confirmed by AFM imaging. After AFM imaging, the tip was brought above the O_s_H and the feedback loop was turned off. The tip was vertically approached on top of the O_s_H and KPFS measurement was performed. We repeat this procedure by changing the vertical tip height and the lateral tip position on top of the O_s_H. As we can see in Fig. 2(b), the bias voltage of the frequency shift jump (*V*_jump_) in KPFS becomes large by reducing the tip height. These results qualitatively demonstrate that further bias voltage is required to reversibly manipulate the configuration between O_s_H−(O_ad_H)^−^−O_s_ and O_s_−(O_ad_H)^−^−O_s_H at a large tip-sample distance. One has to keep in mind that for a non-zero local contact potential difference (LCPD) between tip and sample, even at zero bias voltage there is a finite electric field across the junction^31–33^. Moreover, this LCPD also depends on the tip-sample distance owing to the average effect^31^. Only at compensated LCPD, that is, at *V*_bias_ = *V*_LCPD_ the local electric field vanishes. Although this is very well known from KPFS, it is usually less considered in pure STM experiments. The quantitative assignment of *V*_jump_ is further shown in Fig. 2(c) with more tip-sample distances including *V*_LCPD_, where *V*_jump _− *V*_LCPD_ linearly becomes large by reducing the tip height. Moreover, during the negative bias KPFS, the current was in the noise limit of our current amplifier (see Supplementary Fig. S2). Additionally, the repulsive interaction does not induce manipulation (see Supplementary Fig. S3). These observation leads us to conclude that the dominant role of the manipulation is the local electric field. Based on Fig. 2(c), the local electric field for manipulating hydrogen was estimated using the following equation

$$E = \left( {V_{{\mathrm{jump}}}-V_{{\mathrm{LCPD}}}} \right)/z,$$

where, *V*_jump_ is the bias voltage where the frequency shift jump occurs, *V*_LCPD_ is the local contact potential difference of the upper parabola in each KPFS^31–33^ and *z* is the relative tip-sample distance. Here, we note that the *V*_LCPD_ is obtained by fitting the parabolic function to the upper parabola of each KPFS obtained at different tip height. Figure 2(c) shows the best linear fit with a slope of −5.6 V/nm. The physical meaning of −5.6 V/nm is the threshold of the local electric field between the tip and the sample, which is necessary to induce the transition between O_s_−(O_ad_H)^−^−O_s_H and O_s_H−(O_ad_H)^−^−O_s_. This linear behavior is similar to the experimental evidence for the electric field-stimulated reaction of oxygen adatom^27^ and desulfurization process on metal surface^48^. Estimating the exact value of the local electric field might be challenging because the local electric field will also generally be affected by the local bulk condition, such as Ti interstitial and subsurface defects on this surface^44,51–53^, and the exact tip apex shape.

### Desorption of hydrogen atoms from a rutile TiO_2_ (110) surface

**Fig. 3:**
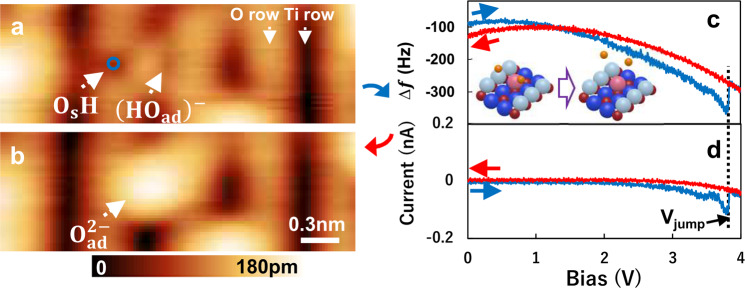
Positive sample bias applied to the O_s_H−(O_ad_H)^−^−O_s_. **a**, **b** AFM images before (**a**) and after (**b**) KPFS manipulation of O_s_H−(O_ad_H)^−^−O_s_. Imaging parameters for (**a**, **b**): constant Δ*f* mode, *V*_bias_ = 0 V and 1.0 × 3.0 nm^2^. **c**, **d** Results of KPFS and tunneling current applied in **a**. The feedback loop was switched off during the measurements, and the sample bias was ramped from zero to a certain positive voltage and then ramped back to zero. The blue and red lines show the forward and backward directions, respectively. The tip position is indicated by a blue circle in (a). The black dotted line serves as a guide for the eye for the *V*_bias_ = *V*_jump_.

To give more insight to the manipulation mechanism, we also performed the positive bias KPFS on top of the O_s_H in O_s_H−(O_ad_H)^−^−O_s_. In the case of field-induced switching mechanisms, the reverse polarity should lead to the opposite perturbation to the potential landscape on the surface^45,46,54–56^. Figure 3(a–d) shows the experimental results of applying a positive bias to the hydrogen atom of O_s_H in O_s_H−(O_ad_H)^−^−O_s_. Figure 3(a) shows an atomically resolved AFM image of O_s_H−(O_ad_H)^−^−O_s_ species prepared by a similar procedure as shown in Fig. 1(a) → 1(f) → 1(b). After AFM imaging (Fig. 3(a)), the tip was moved on top of the O_s_H and the bias was ramped from zero to a certain positive voltage and then back to zero. Figure 3(c) shows the KPFS applied between Fig. 3(a) and (b). As we can see in Fig. 3(c), the two parabolas appear accompanied by the *V*_LCPD_ shift and *V*_jump_ at +3.8 V. The *V*_LCPD_ of blue and red curves in Fig. 3(c) are +0.5 V and +1.1 V. The corresponding *V*_LCPD_ shift to larger bias voltage after jump indicates the reduction of the positive charge under the tip^31–33^. Figure 3(b) shows the AFM image of the same scan area at 0 V obtained immediately after the bias voltage back to zero, showing that the two black spots corresponding to the hydrogen atoms disappeared and O_ad_^2−^ appear, suggesting that the two positively charged hydrogens desorbed from the surface. This is in line with the positive *V*_LCPD_ shift shown in Fig. 3(c). Interestingly, as shown in Fig. 3(d), we also found that significant tunneling current flow during the positive bias KPFS. The absolute tunneling current of O_s_H and oxygen row (O_s_) at *V*_jump_ are 0.105 nA and 0.039 nA. This observation agrees with the previous STM results that O_s_H flows larger tunneling current than O_s_ under the typical positive bias condition^1–6,10–22,25,26^. The observed tunneling current with the positive bias KPFS is contrary to that of negligible tunneling with negative bias KPFS (see Supplementary Fig. S2), thus the dominant contribution to the hydrogen desorption may be induced by the tunneling current^3,10^. Hence, suggesting that the local electric field has a dominant role in the lateral manipulation of the hydrogen atom on this surface.

### Manipulation mechanism of hydrogen atoms on a rutile TiO_2_ (110) surface

**Fig. 4:**
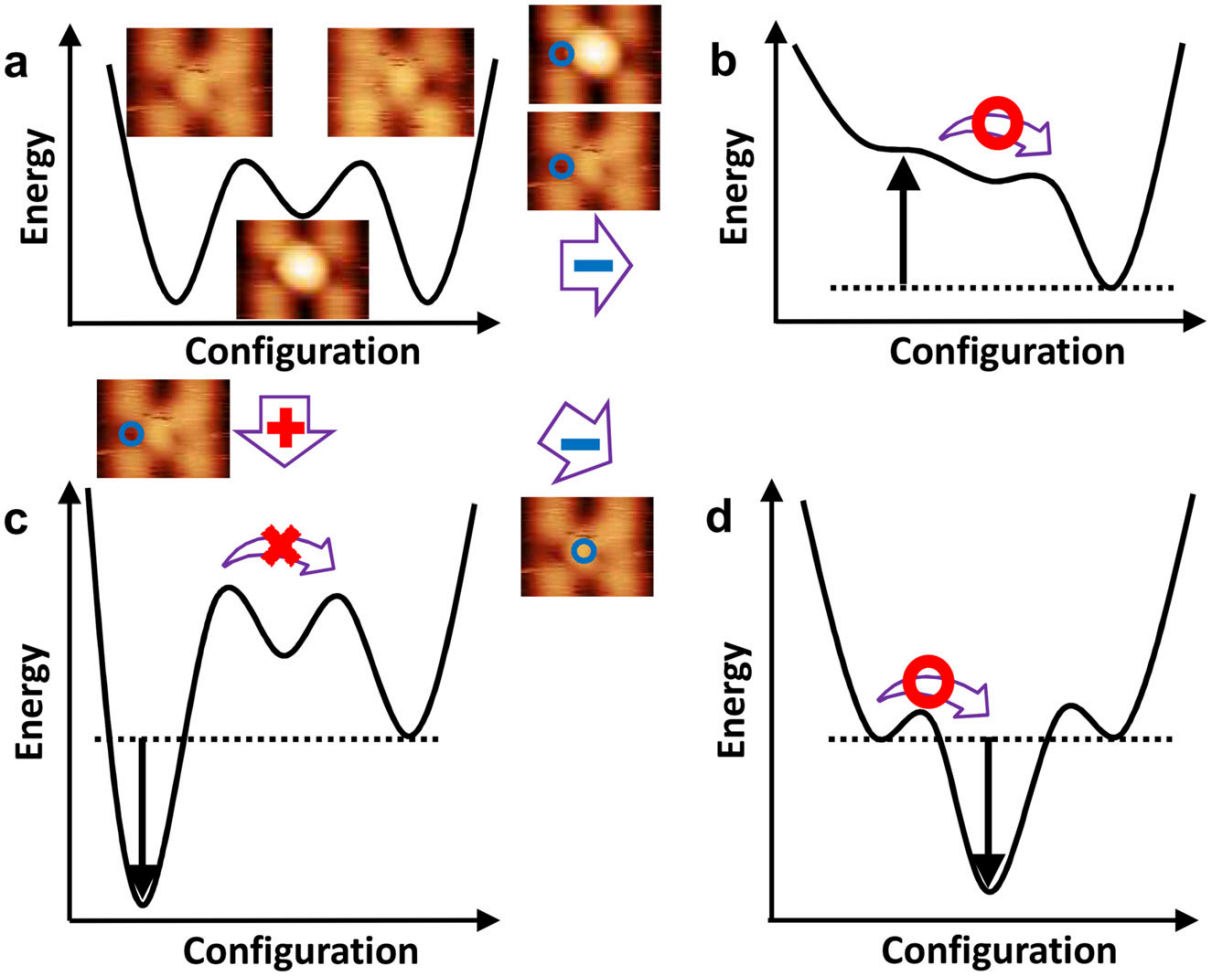
Schematic potential energy landscape of manipulation. **a** Schematic potential energy curves of the triple well in which the triple minima correspond to O_s_H−(O_ad_H)^−^−O_s_, O_s_H−O_ad_^2−^−O_s_H and O_s_-(O_ad_H)^−^-O_s_H, as indicated by the images in **a**. **b**–**d** Schematic potential energy curves depending on the lateral position of the tip and applying a positive and negative bias between tip and sample. The black arrow indicates the perturbation of the potential landscape owing to the electric field. The real potential energy for describing the manipulation is much more complicated, and many factors may need to be considered, such as the local adsorbate charge, subsurface effect and the precise shape of the tip. The tip position is indicated by a blue circle in the AFM images.

The reaction among three atomic structures can be commonly described by a multiple umbrella potential well, as shown in Fig. 4(a)^45,54–56^. The three energy minima define the vibrational ground states of the O_s_H−(O_ad_H)^−^−O_s_, O_s_H−O_ad_−O_s_H and O_s_−(O_ad_H)^−^−O_s_H are separated by a potential barrier representing the activation energy required to induce the configuration change. Our DFT calculation in Supplementary Table S1 shows that the O_s_−(O_ad_H)−O_s_H is generally more energetically favorable than O_s_H−O_ad_−O_s_H. Based on these results, inducing the local electric field in a specific site with respect to the atomic configuration deforms the potential energy landscape of three different configurations in a characteristic manner and thus changes the configuration, as shown in Fig. 4(b–d)^45,54–56^. This can be achieved by changing the polarity of the bias voltage between the tip and sample, and the lateral position of the tip. In the case of reversible manipulation between O_s_−(O_ad_H)^−^−O_s_H and O_s_H−(O_ad_H)^−^−O_s_ (Fig. 4(a) and (b)), the tip was placed on top of the O_s_H. As is discussed in Fig. 1(a–i), we observed a direct transition between the bistable state, yielding a local electric field lifting up the potential energy of the ground state of the O_s_H−(O_ad_H)^−^−O_s_ (Fig. 4(a)→4(b)). In this process, the sufficient local electric field of −5.6 V/nm in our experiment should be enough to overcome such a barrier, ~0.22 eV reported by Tan et al.^3^. On the contrary, applying positive bias on top of the O_s_H, lower the potential energy of the ground state of the O_s_H−(O_ad_H)^−^−O_s_ as shown in Fig. 3(a)−(d), hence result in further stabilization of the O_s_H−(O_ad_H)^−^−O_s_ (Fig. 4(a)→4(c)). However, the sufficient tunneling current leads to the desorption of the hydrogen atom, not the lateral hopping of hydrogen atom on this surface as again shown in Fig. 3(a)–(d). This desorption mechanism might be related to the vibrational excitation by inelastic tunneling, which is previously reported by Acharya et al.^10^. Consider now applying a negative bias to the oxygen atom corresponding to the tip positioned at the center of the O_s_H−(O_ad_H)^−^−O_s_ (Fig. 4(a) →4(d)). It is seen after the bias was swiped in the negative direction, that one of the hydrogen atoms directly underneath the tip moved toward the oxygen row, result in O_s_H−O_ad_^2−^−O_s_H (Fig. 1(d) → 1(i) → 1(e)). As shown in Fig. 4(a) →4(d), this can be realized by lowering the potential energy of O_s_H−O_ad_^2−^−O_s_H, which is similar to the mechanism of the reversible manipulation between O−(O_ad_H)^−^−O_s_H and O_s_H−(O_ad_H)^−^−O. Our DFT calculation also supports that an electric field up to −0.5 V/nm would significantly stabilize O_s_H−O_ad_−O_s_H (Supplementary Table S1).

### Force spectroscopy of water on a rutile TiO_2_ (110) surface

**Fig. 5:**
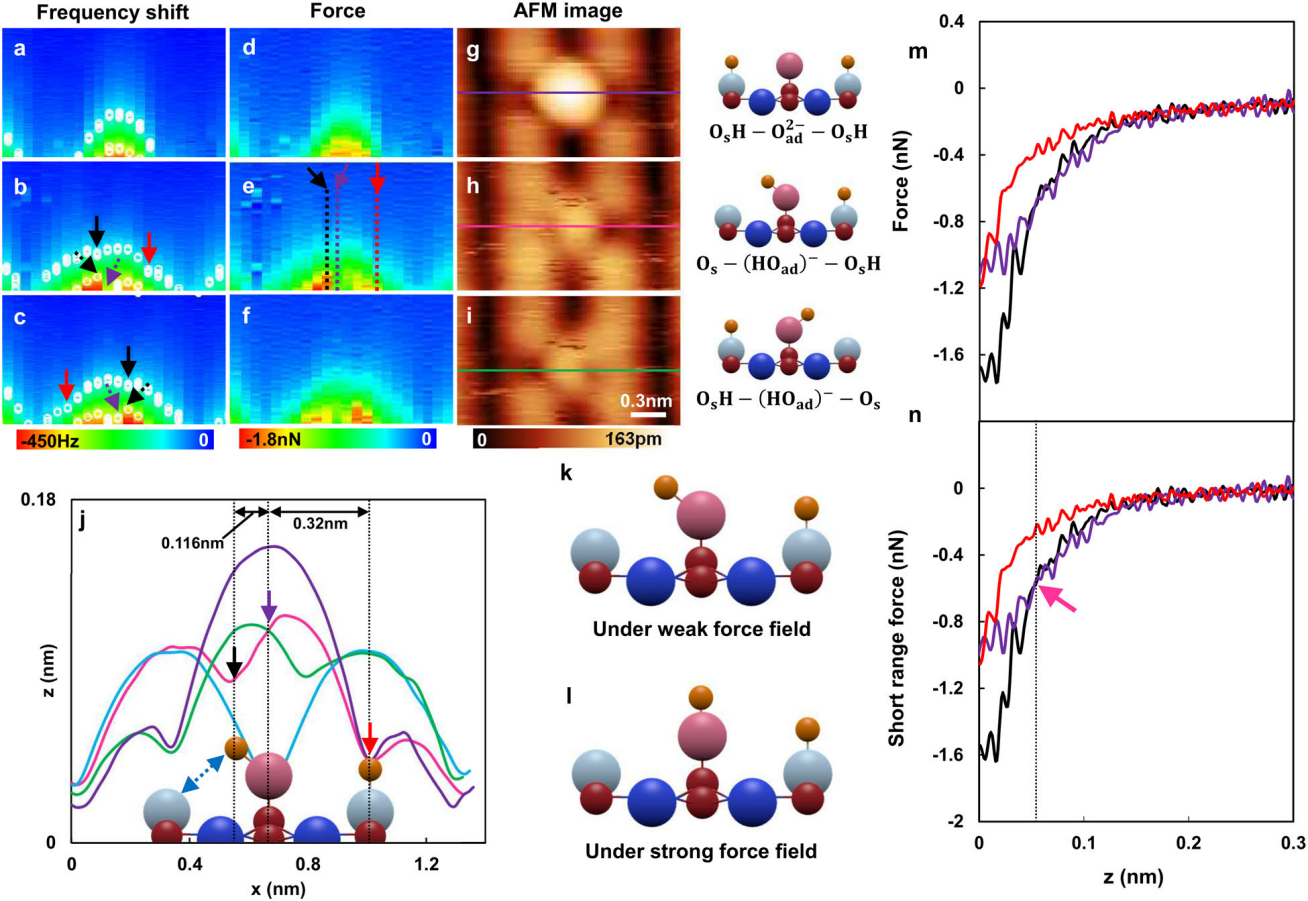
Exploration of the atomic configuration of O_s_H−O_ad_^2−^−O_s_H, O_s_−(O_ad_H)^−^−O_s_H, O_s_H−(O_ad_H)^−^−O_s_. **a**–**f** ∆*f*(z) (**a**–**c**), and *F*(*z*) (**d**–**f**) maps for approach measured along the $[1\bar 10]$ direction. The white circle in **a**–**c** indicates the constant frequency of −145 Hz and −345 Hz. The black and red solid arrows in **a**–**c** indicate the slight dip positions in a constant frequency of −145 Hz. The black and purple dotted arrow indicates the local maximum and minimum of −345 Hz. Spectroscopy parameters for **a**–**f**: *V*_bias_ = 0 V, 0.24 × 1.60 nm^2^. **g**–**i** AFM images of O_s_H−O_ad_^2−^−O_s_H, O_s_−(O_ad_H)^−^−O_s_H, and O_s_H−(O_ad_H)^−^−O_s_. Imaging parameters for **g**–**i**: 1.0 × 1.3 nm^2^. **j** Line profiles measured for the three species and substrate: O_s_H−O_ad_^2−^−O_s_H, O_s_−(O_ad_H)^−^−O_s_H, O_s_H−(O_ad_H)^−^−O_s_ and O_s_−O_ad_^2−^−O_s_. Purple, O_s_H−O_ad_^2−^−O_s_H; pink, O_s_−(O_ad_H)^−^−O_s_H; green, O_s_H−(O_ad_H)^−^−O_s_; light-blue, O_s_−O_s_. The schematic of the surface structure is superimposed in the graph. The blue dotted arrow indicates the interaction between the hydrogen atom and the oxygen row. The black and red solid arrows indicate the dip positions in the pink curve. The purple solid arrow indicate the middle of the structure. The black dotted lines are guides for the eyes. The positions of the line profiles in **j** are shown in different colors in **g**–**i**. **k**–**l** Schematic structure of O_s_−(O_ad_H)^−^−O_s_H under weak and strong force field. **m**, **n** *F* (*z*) and *F*_SR_ (*z*) curves obtained on top of the O_s_−(O_ad_H)^−^−O_s_H. The positions of the curves are indicated by the arrows, and the dotted lines inside **j** and **e** by different colors. The pink arrow in **n** indicates the cross point between black and purple curves.

To consider the precise three atomic configurations of O_s_H−O_ad_^2−^−O_s_H, O_s_−(O_ad_H)^−^−O_s_H and O_s_H−(O_ad_H)^−^−O_s_, now we analyze the hydrogen configurations of three manipulation outcomes based on AFM images and force field images. Figure 5(a–c), (d–f), and  (g–i) show the ∆*f*(z) mapping, *F*(*z*) mapping and AFM images obtained on top of the O_s_H−O_ad_^2−^−O_s_H, O_s_−(O_ad_H)^−^−O_s_H and O_s_H−(O_ad_H)^−^−O_s_. The Δ*f*(*z*) mapping experiment was performed as follows. The lateral tip position was fixed at the side of the oxygen row and the Δ*f*(*z*) was recorded over O_s_H−O_ad_^2−^−O_s_H, including a long-range force from the TiO_2_ substrate. Then, the tip was placed on top of the hydrogen atom and KPFS measurement was performed. We repeat this procedure by manipulating among the three configurations of O_s_H−O_ad_^2−^−O_s_H, O_s_−(O_ad_H)^−^−O_s_H and O_s_H−(O_ad_H)^−^−O_s_. Notably, *F*(*z*) was obtained from Δ*f*(*z*) using Sader Jarvis method^57^. Fig. 5(j) shows the line profiles measured on top of the O_s_H−O_ad_^2−^−O_s_H, O−(O_ad_H)^−^−O_s_H and O_s_H−(O_ad_H)^−^−O_s_ configurations. The positions of the line profiles are shown in Fig. 5(g–i) in different colors in the images. In Figure 5(j), we observe that both the O_s_−(O_ad_H)^−^−O_s_H and O_s_H−(O_ad_H)^−^−O_s_ configurations have symmetric line profiles, and most importantly, the two hydrogen positions in the O_s_−(O_ad_H)^−^−O_s_H configuration indicated by the black and red arrows. Focusing on the light-blue and pink curves, the hydrogen atom on the right (red arrow) has the same position as the oxygen row (the local maxima position of Z in the oxygen row is generally the same as the local minima position of the dip). However, the hydrogen atom on the left (black arrow) is slightly tilted to the left from the center of O_ad_^2−^. The distances from the center of O_ad_^2−^ to these dips were 320 pm and 116 pm, respectively. Notably, the distance of 320 pm is the half distance between the oxygen row^1^, which indicates the reliable lateral resolution of the contrast in the AFM images Fig. 5(g–i). From these observations, we expect that the hydrogen atom on left will strongly be perturbed from the top and orient to the left owing to the creation of a hydrogen bond with the nearest neighbor oxygen row (as shown by the blue dotted arrow in Fig. 5(j)). On the other hand, the hydrogen atom on the right has similar interactions from (O_ad_H)^−^ and the nearest neighbor oxygen row; therefore, a too small tilt was observed (limited by the convolution of the tip apex shape and sample structure). This assertion is also supported by the similar height of (O_ad_H)^−^ and the nearest neighbor oxygen row. The equivalent net charge of (O_ad_H)^−^ and the nearest neighbor oxygen row result in a similar topographical height owing to the contrast mechanism of the tip in hole mode^9,30,34^. Moreover, this off-center orientation of the hydrogen bond in Fig. 5(j) are nicely demonstrated in our DFT calculation shown in Supplementary Figure S4(d), and also the theoretical prediction in Tan et al.^3^ and Du et al.^5^ studies. Hence, give us extra confidence to conclude that the hydrogen atom on the right has similar interactions from (O_ad_H)^−^ and the nearest neighbor oxygen row. Here, we should note that one would expect a small influence of the possible tilting of the hydrogen atom under the attractive force interaction. Because, for example, the AFM imaging shows different bond lengths and atom positions in C_60_ when changing the *Z* distance^39^. Therefore, we perform the frequency shift mapping as shown in Fig. 5(a–c). The overall values of ∆*f*(*z*) decrease by reducing the tip height. To allow easy visualization, we draw a constant frequency shift value of −145 Hz and −345 Hz by the circle in Fig. 5(a–c), respectively. Importantly, the value of −145 Hz is the conventional value for stable AFM imaging during the experiment. Focusing on the constant frequency shift value of −145 Hz in Fig. 5(b, c), we also found a similar characteristic of the off-center orientation of the hydrogen bond as shown in Fig. 5(j). The two hydrogen atoms identified in Fig. 5(j) were identically indicated by the black and red solid arrows in Fig. 5(b, c). This finding is in perfect agreement with the contrast mechanism of the tip in the hole mode, which a positively charged tip gives rise to additional attractive interaction with the negatively charged oxygen atom on the surface, causing a larger negative frequency shift, and the oxygen atom appears bright^9,30,34^. On the other hand, the position of the hydrogen atom is “less negative” than the oxygen atom, which gives them a slight dark contrast. Remarkably, when the tip approaches very close to the surface, the local maximum of ∆*f*(*z*) appears on top of the hydrogen atom, which is indicated by the black dotted arrow of the constant frequency of −345 Hz in Fig. 5(b, c). This finding also perfectly agree with the contrast inversion expected in the small tip-sample distance, which, the strong attractive force field induce a displacement of the hydrogen atom, gives rise to the screening of the underlying oxygen atoms, and the overall interaction results in a convolution between the positive tip apex, positive hydrogen, and the negative oxygen atom^30,34^ as shown in Fig. 5(k, l). The displaced hydrogen atom tends to relocate at the center of the oxygen atom, which is nicely demonstrated by the local minima of the ∆*f*(*z*) mapping in Fig. 5(b, c) as indicated by the purple dotted arrow. Therefore, the AFM contrast obtained at −145 Hz has a small influence on the possible tilting of the hydrogen atom under the AFM imaging. Figure. 5(d–f) show the *F*(z) mapping obtained from Fig. 5(a–c). The overall values of *F*(z) decrease by reducing the tip height, owing to the increasement of the attractive force. In Fig. 5(m) and (n), the component of the force curve between tip and sample at different locations are further evaluated from the short range force. The positions of the curves are indicated by the solid arrows, and the dotted lines inside Fig. 5(e) and (j) by different colors. The short range force commonly offers an atomic resolution in AFM. On the other hand, long-range force offers background force acting on the tip at a relatively far distance from the surface, such as long-range van der Waals force and long-range electrostatic force^58,59^. The long-range dominant region of the ∆*f* (z) curves, which we defined as *z* > 0.3 nm, was fitted into the inverse-power function of *z*^−s^ ^58,59^. The short range part of ∆*f* (*z*) was obtained by subtracting the long-range part of ∆*f* (*z*) from the raw ∆*f* (*z*) curve. Then, the short range part of ∆*f* (*z*) curve was numerically converted to *F*_SR_ (*z*) using the Sader Jarvis method^57–59^. In Fig. 5(m), the three curves of *F* (*z*) generally decrease by reducing tip-sample distance, which indicates the attractive tip-sample interaction. Especially in Fig. 5(n), we found that *F*_SR_ (*z*) has a similar tendency with *F* (*z*). Hence, the atomic contrast of Fig. 5(g–i) and *F* (*x*, *z*) in Fig. 5(d–f) are dominantly governed by the short range forces. When the contrast inversion occur in the *F*_SR_, the attractive short range force acting on the tip was about *F*_SR_ = −0.6 nN, indicated by the pink arrow in Fig. 5(n). Suppose that the hydrogen relocation occur nearly at the tip-sample distance where the contrast inversion occur in the *F*_SR_, the results shown in Fig. 5(n) indicate that the hydrogen bond can be stabilized in this configuration without rearrangement, at the range of −0.6 nN < *F*_SR_. In the DFT calculation shown in Supplementary Fig. S5, the force required for the rearrangement of O_ad_H from tilted geometry to upright is estimated to be around 0.440 nN, which is smaller than experimentally measured 0.6 nN. This difference additionally propose that the rearrangement of the hydrogen atom is presumably induced by the attractive force of tip background short range van der Waals interaction or tip dipole, dominates the tip-sample interaction^60–62^. Compared to the other works, the magnitude of *F*_SR_ = −0.6 nN is quantitatively larger than the lateral or vertical force for displacing a physisorbed CO molecule on metal surface^38^. This is generally in line with the physical aspect of the hydrogen-oxygen atom interaction that the hydrogen bond is generally stronger than the van der Waals interaction^40,63,64^. Hence, suggesting that the deprotonated configurations of O_s_−(O_ad_H)^−^−O_s_H and O_s_H−(O_ad_H)^−^−O_s_ are significantly stable under the specific force field.

### Control of single hydrogen atom on a rutile TiO_2_ (110) surface

**Fig. 6:**
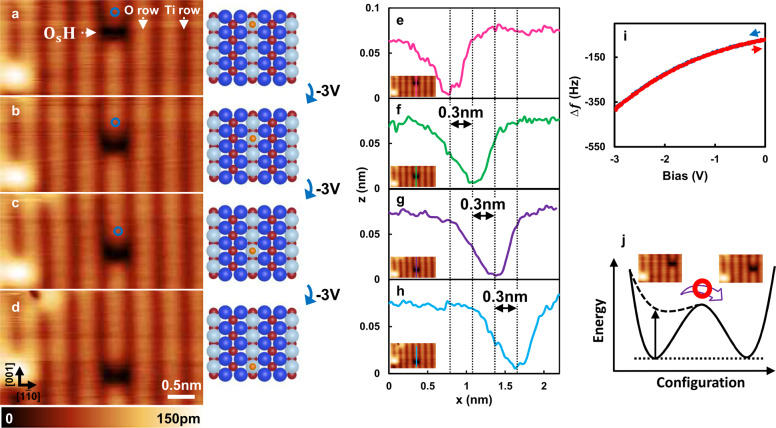
Lateral manipulation of hydrogen atom along the [001] direction using the KPFS. **a** AFM image of O_s_H defect on the rutile TiO_2_ surface. **b**–**d** Consecutive AFM images obtained after KPFS on top of the oxygen row. The tip position is indicated by a blue circle in the images (**a**–**c**). Imaging parameters for **a**–**d**: constant Δ*f* mode, *V*_bias_ = 0 V, **a**–**c** 2.1 × 5.2 nm^2^, and **d** 2.8 × 5.2 nm^2^. **e**–**h** Line profiles measured for the O_s_H defect and substrate. The black dotted lines indicate the position of the hydrogen atoms. The positions of the line profiles are shown in different colors in the inset images. **i** Typical KPFS performed during the lateral manipulation shown in **a**–**c**. The feedback loop was switched off during the KPFS measurements, and the sample bias was ramped from 0 V to −3 V and then back to 0 V. Naturally, no jump in frequency shift was observed during the voltage ramp. **j** Schematic potential energy curves depending on the lateral position of the OH defect and applying a negative bias between tip and sample. The black arrow indicates the perturbation of the potential.

Finally, we demonstrate the capability of negative bias KPFS for the lateral manipulation of O_s_H defect on a rutile TiO_2_ surface using an individual single O_s_H defect, because this O_s_H defect is also the most fundamental atomic feature on this surface^1^ and known to provide a critical role in photocatalysis at tremendous condition^1–23^. Figure 6(a) shows an atomically resolved AFM image of a rutile TiO_2_ (110) surface partially exposed to oxygen at room temperature obtained using a hole mode tip^9,30,34^. The black spot can be observed in Fig. 6(a) that corresponds to the O_s_H defect^9,30,34^. Notably, an oxygen adatom can be observed on the lower left side as a bright spot. Next, we demonstrate that this O_s_H defect can be precisely manipulated along the [001] direction by KPFS. After AFM imaging (Fig. 6(a)), the tip was brought slightly to the upper side of the O_s_H defect, and the bias was ramped from zero to a certain negative voltage and then back to zero (Figure 6(i)). Fig. 6(b) shows the AFM image of the same scan area obtained at 0 V immediately after the bias back to zero, showing that the black spots that correspond to O_s_H defect moved one-lattice distance toward the [001] direction from its initial position. As shown in Fig. 6(j), Li et al.^14^ has reported the H diffusion along the [001] direction with an energy barrier of ~1.29 eV. This sufficient large energy barrier is supposed to prevent spontaneous diffusion under the 78 K. Hence, inducing the local electric field near to the hydrogen atom deforms the potential energy landscape, and result in the lateral hopping of the hydrogen along the [001] direction, as shown by the black dotted line in Fig. 6(j). Interestingly, in this process, we also found that the bias voltage of about *V*_bias_ ≦ −3.0 V is required to hop the hydrogen along the [001] direction (Fig. 6(i)). This finding nicely agrees with the theoretical aspects that the larger bias voltage is required to overcome such a barrier, ~1.29 eV^14^. Notably, we previously found that the positive bias will easily induce the desorption of the hydrogen atom on this surface^9^. Figure 6(c) and (d) show the reproducibility of this manipulation, which indicates that the hydrogen atom can be fully manipulated along the [001] direction without the atom being desorbed. The quantitative assignment of these manipulations can be performed by measuring the line profile on top of the hydrogen atom as shown in Fig. 6(e–h). We found that the displacement of each hydrogen atom is 0.3 nm, which is in perfect agreement with the nearest neighbor distance between the two oxygen atoms in the oxygen row^1^. Therefore, the hydrogen atom can be precisely controlled at the single atomic level using negative bias KPFS.

In summary, we have demonstrated the lateral manipulation of hydrogen atom on the rutile TiO_2_ (110) surface by low-temperature AFM and KPFS. We succeeded in the reliable control and characterization of a hydrogen atom on top of the three different outcomes of O_s_H−O_ad_^2−^−O_s_H species by using a functionalized tip in hole mode with the KPFS manipulation. The force mapping with an atomic resolution allowing us to preciously determine the hydrogen position; interestingly, one hydrogen atom was tilted forward and another was straight. We believe that the achievement of our large body of work intrinsically provides the opportunity to understand the mechanochemical process of reactive oxygen species, the hydrogen atom and water species, naturally the world’s most important chemical species, on an oxide surface.

## Methods

### Experimental details

The experiments were carried out using a low-temperature ultrahigh vacuum AFM system. The deflection of the cantilever was measured using the optical beam deflection method. The base pressure was lower than 5.0 × 10^−11^ Torr. The temperature of the AFM unit was kept at liquid nitrogen temperature (78 K). The AFM measurements were performed in the frequency modulation (FM) detection mode. The atom tracking method was used to compensate for the thermal drift between the tip and surface during the measurements. The dc bias voltage was applied to the sample. The AFM imaging was performed using constant Δ*f* mode at *V*_bias_ = 0 V to avoid the tunneling current to flow. The cantilever was oscillated at resonance frequency keeping the oscillation amplitude constant. We used iridium (Ir)-coated Si cantilever (Nanosensors SD-T10L100, *f*_0_ = 800 kHz, *A* = 500 pm, k = 1500 N/m). Metal Ir tips provide stable AFM imaging compared to the bare Si tip. The tip was initially annealed to 600 K and then cleaned by Ar^+^ sputtering to remove the contamination before experiments. The rutile TiO_2_ (110)-(1 × 1) sample was prepared by sputtering and annealing to 900 K in several cycles. The sample was exposed to oxygen at room temperature for ~0.5 L and then transferred to the measurement chamber precooled to 78 K. The O_s_H groups on the TiO_2_(110) surface were spontaneously created from the dissociation of water molecules (from a background residual vacuum) over oxygen vacancy sites by transferring hydrogen atoms to neighboring oxygen bridge sites^1^. We modify the tip apex by gently poking the tip into the surface using the controlled force distance spectroscopy, until reaching to the sharp tip in hole mode (positively terminated tip). Notably, we can distinguish O_s_H from the oxygen vacancies using the previous results^9^.

### Calculation method

The DFT calculations were carried out using the CP2K Quickstep package^65^ and the PBE0-TC-LRC-ADMM hybrid density functional^66,67^, containing 20% HFX exchange, which was truncated at a distance of 2.5 Å. The cutoff of the finest real-space integration grid is 400 Ry. The primary basis set is MOLOPT^68^, of DZVP quality for Ti and TZV2P quality for O, with corresponding GTH pseudopotentials^69,70^. The auxiliary Gaussian basis for the ADMM method was cFIT11 for Ti and cFIT3 for O. The dispersion interactions were considered within the Grimme D3 method^71^. The force convergence criterion used for geometry relaxations was 0.01 eV/Å. The TiO_2_ (110) surface was constructed using slab model consisting of six atomic layers and cell parameters from bulk calculation. The external electric field was simulated by introducing a saw-shaped periodic electrostatic potential along *z*-axis. In the evaluation of surface geometry under external load, external force was applied on the H atom along *z*-axis.

## Supplementary information

Supplementary Information

## Data Availability

The data supporting the findings of this study are available from the corresponding authors upon reasonable request.
