# Supplementary material for: Single hydrogen atom manipulation for reversible deprotonation of water on a rutile TiO2 (110) surface
Source: Commun Chem. 2021 Jan 19;4:5. doi: 10.1038/s42004-020-00444-4 (PMC9814442; doi:10.1038/s42004-020-00444-4)
Supplement: Supplementary file 1 — Supplementary Information [file 42004_2020_444_MOESM1_ESM.pdf]

Supporting information to

Single hydrogen atom manipulation for reversible deprotonation of water on a rutile TiO_2 (110) surface

Yuuki Adachi[†], *Hongqian Sang*[‡], *Yasuhiro Sugawara*[†], *Yan Jun Li*[†]*

[†]Department of Applied Physics, Osaka University, 2-1 Yamadaoka, Suita, Osaka 565-0871, Japan

[‡]Institute for Interdisciplinary Research, Jianghan University, Wuhan 430056, China

Description of Additional Supplementary Files

Name: Supplementary Figure S1
Description: Large area AFM image of O_sH-O_{ad}^{2-}-O_sH species on rutile TiO_2 (110) surface.

Name: Supplementary Figure S2
Description: Simultaneously obtained current signal with negative bias KPFS.

Name: Supplementary Figure S3
Description: $\Delta f(z)$ curve obtained on top of the O_sH in O_sH-$(O_{ad}H)^-$-O_s.

Name: Supplementary Figure S4
Description: DFT optimized geometries of O_sH-O_{ad}^{2-}-O_sH (a-c) and O_s-$(O_{ad}H)^-$-O_sH.

Name: Supplementary Figure S5
Description: Geometries of O_s-$(O_{ad}H)^-$-O_sH under external vertical load applied on H atom from $(O_{ad}H)^-$.

Name: Supplementary Table S1
Description: DFT energy of O_sH-O_{ad}^{2-}-O_sH relative to that of O_s-$(O_{ad}H)^-$-O_sH under external electric field.

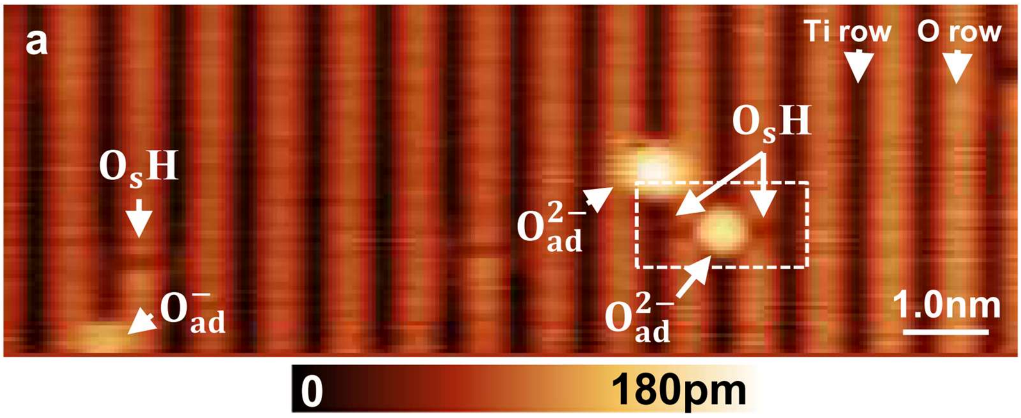

Supplementary Figure S1 Large area AFM image of O_sH-O_{ad}^{2-}-O_sH species on rutile TiO_2 (110) surface. (**a**) The large area of AFM image obtained on top of the rutile TiO_2 (110) surface was exposed by oxygen at room temperature. The tip is hole mode. The bright row is the oxygen row and the dark row is Ti row[1-4]. Black spots that correspond to O_sH defects can be observed on the oxygen row[1-4]. Different types of stable bright features above the Ti rows, most importantly the bright O_{ad}^{2-} and less bright O_{ad}^{-}, are seen in (a) [3, 4], Notably, O_{ad}^{2-} is the most stable oxygen species on the rutile TiO_2 surface[4]. Imaging parameters: constant Δf mode, V_{bias} = 0 V, 3.5 × 10nm^2. The white dotted box indicates O_sH-O_{ad}^{2-}-O_sH. Moreover, we previously used simultaneous STM and AFM mode to investigate the O_sH defects[5].

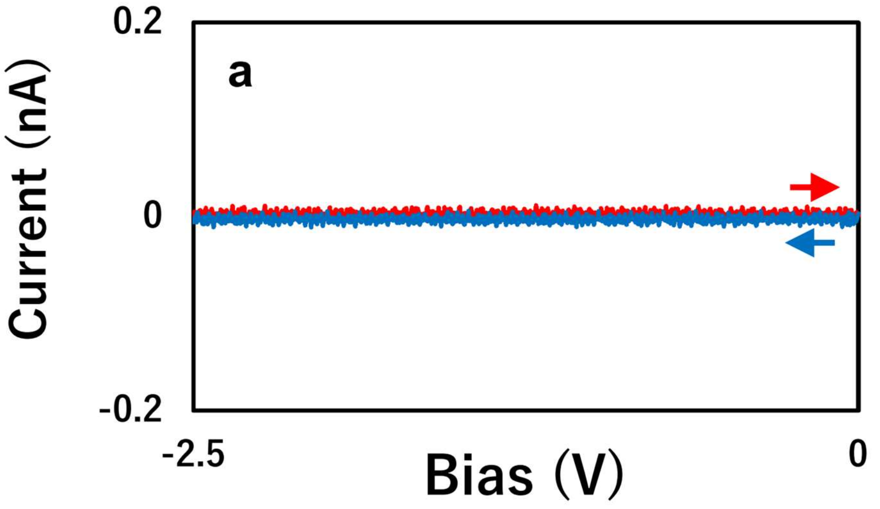

Supplementary Figure S2 Simultaneously obtained current signal with negative bias KPFS. (**a**) Simultaneously obtained current signal with KPFS shown in Fig. 2(b) at 0 pm.

Supplementary Figure S2(a) shows simultaneously obtained current signal with KPFS shown in Fig. 2(b) at 0 pm in the main text. Notably, we also did not observe significant current changes during the negative KPFS. This is because the tip-sample distance is enough large and our TiO_2 sample has ~3.0 eV bandgap roughly from 0.6 and −2.6 V, which is related to the conduction and valence bands[6]. This bandgap size is in good agreement with the previous reports[1].

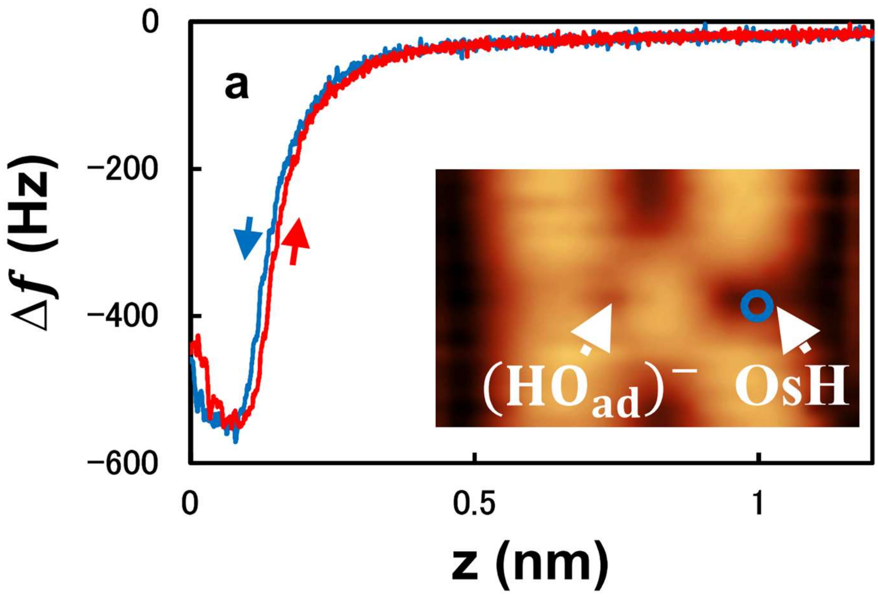

Supplementary Figure S3 $\Delta f(z)$ curve obtained on top of the O_sH in O_sH-$(O_{ad}H)^-$-O_s. (**a**) $\Delta f(z)$ curve measured on top of the O_sH in O_sH-$(O_{ad}H)^-$-O_s. The blue and red curves show the forward and backward directions, respectively. Spectroscopy parameters: V_{bias} = 0 V. Inset image shows the AFM image of O_sH in O_sH-$(O_{ad}H)^-$-O_s. Imaging parameters: constant Δf mode, V_{bias} = 0 V, 1.2 × 1.3 nm^2.The tip position is indicated by a blue circle in the AFM image.

Supplementary Figure S3(a) shows $\Delta f(z)$ curve obtained on top of the O_sH in O_sH-$(O_{ad}H)^-$-O_s. The $\Delta f(z)$ shows a monotonic decrease in the attractive regime at 0.07 nm < z < 1.20 nm, and an increase in the repulsive regime at 0.00 nm < z < 0.07 nm. However, there is no clear hysteresis between the forward and backward curves (blue and red curves

in Supplementary Figure S3(a)). Therefore, a repulsive interaction does not play a crucial role in the manipulation between O_s-$(O_{ad}H)^-$-O_sH and O_sH-$(O_{ad}H)^-$-O_s.

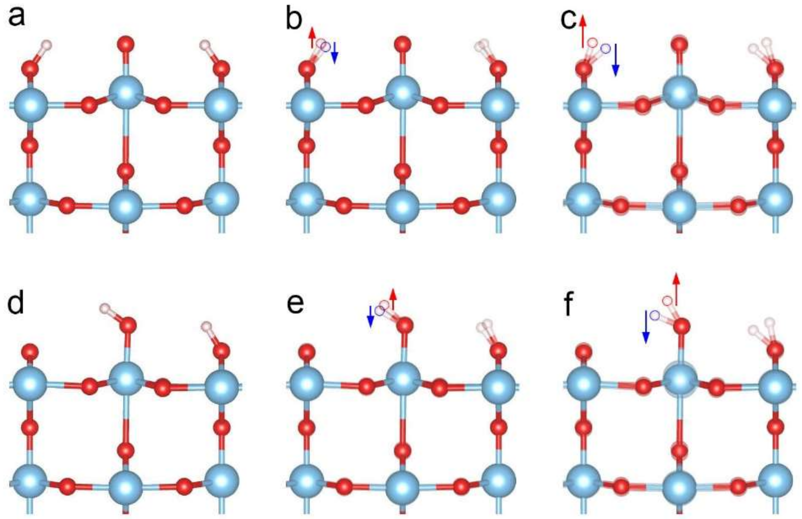

Supplementary Figure S4 DFT optimized geometries of O_sH-O_{ad}^{2-}-O_sH (a-c) and O_s-$(O_{ad}H)^-$-O_sH. (d-f) under the influence of external electric field calculated by CP2K. The electric field strength is ± 2 V/nm in (b)(e) and ± 5 V/nm in (c)(f), and direction of the field is marked by red (positive sample bias) or blue (negative sample bias) arrows.

As shown in Supplementary Figure S4, the H atom behaves like a positively charged proton under the influence of external electric field as expected. And the displacement of H atom in simulation is noticeable when the electric field strength is comparable to experimental ones.

Electric Field (V/nm)	0.5	0.2	0	-0.2	-0.5
Energy Difference (eV)	0.450	0.289	0.187	0.131	0.047

Supplementary Table S1 DFT energy of O_sH-O_{ad}^{2-}-O_sH relative to that of O_s-$(O_{ad}H)^-$-O_sH under external electric field.

DFT simulation shows O_s-$(O_{ad}H)^-$-O_sH is energetically more stable than O_sH-O_{ad}^{2-}-O_sH, and the energy penalty of later geometry becomes much smaller when negative electric field is applied. Hence, the transition between O_s-$(O_{ad}H)^-$-O_sH and O_sH-$(O_{ad}H)^-$-O_s could be facilitated by a negative bias.

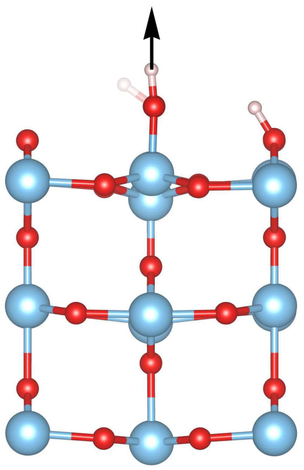

Supplementary Figure S5 Geometries of O_s-$(O_{ad}H)^-$-O_sH under external vertical load applied on H atom from $(O_{ad}H)^-$. The DFT energy increase between upright and tilted geometries demonstrated here is 0.102 eV, and the corresponding displacement of H is 0.071 nm laterally and 0.031 nm vertically.

The geometry of O_s-$(O_{ad}H)^-$-O_sH under external force is also simulated. The vertical force required for the rearrangement of $O_{ad}H$ from tilted geometry to upright is estimated to be around 2.74 eV/nm or 0.44 nN, which is smaller than experimentally measured attractive force of 0.6 nN between tip and sample. The external force is simply applied on the H atom in simulation, while in experiment the situation is more complex. In AFM operated in hole mode, the tip is positively charged and repulsive electric force above H is expected, while attractive force of short range vdW interaction[7,8], presumably dominates the interaction.

References

1. Diebold, U. The Surface Science of Titanium Dioxide. *Surf. Sci. Rep.* **2003**, *48*, 53-229.

2. Wen, H. F.; Adachi, Y.; Zhang, Q.; Miyazaki, M.; Sugawara, Y.; Li, Y. J. Identification of Atomic Defects and Adsorbate on Rutile TiO_2 (110)-(1×1) Surface by Atomic Force Microscopy. *J. Phys. Chem. C* **2019**, *123*(42), 25756-25760.

3. Adachi, Y.; Wen, H. F.; Zhang, Q. Z.; Miyazaki, M.; Sugawara, Y.; Sang, H. Q.; Brndiar, J.; Kantorovich, L.; Štich, I.; Li, Y. J. Tip-Induced Control of Charge and Molecular Bonding of Oxygen Atoms on the Rutile TiO_2 (110) Surface with Atomic Force Microscopy. *ACS Nano* **2019**, *13*, 6917-6924.

4. Zhang, Q. Z.; Li, Y. J.; Wen, H. F.; Adachi, Y.; Miyazaki, M.; Sugawara, Y.; Xu, R.; Cheng, Z. H.; Brndiar, J.; Kantorovich, L.; Štich, I. Measurement and Manipulation of the Charge State of Adsorbed Oxygen Adatom on Rutile TiO_2(110)-1×1 Surface by nc-AFM and KPFM. *J. Am. Chem. Soc.* **2018**, *140*, 15668-15674.

5. Zhang, Q.; Wen, H. F.; Adachi, Y.; Miyazaki, M.; Sugawara, Y.; Xu, R.; Cheng, Z. H.; Li, Y. J. Characterization and Reversible Migration of Subsurface Hydrogen on Rutile TiO_2 (110) by Atomic Force Microscopy at 78 K. *J. Phys. Chem. C* **2019**, *123*, 22595-22602.

6. Adachi, Y.; Sugawara, Y.; Li, Y. J. Remotely Controlling the Charge State of Oxygen Adatoms on Rutile TiO_2 (110) Surface using Atomic Force Microscopy. *J. Phys. Chem. C* **2020**, 124, 22, 12010-12015

7. Schwarz, A.; Köhler, A.; Grenz, J.; Wiesendanger, R. Detecting the Dipole Moment of a Single Carbon Monoxide Molecule. *Appl. Phys. Lett.* **2014**, 105, 011606.

8. H. Hamaker, *Physica* **1937**, 4, 1058.
